# Supplementary material for: Predicting and improving diagnosis of tuberculosis outcomes in South Africa using machine learning techniques
Source: PLOS Glob Public Health. 2025 Nov 7;5(11):e0004674. doi: 10.1371/journal.pgph.0004674 (PMC12594327; doi:10.1371/journal.pgph.0004674)
Supplement: S1 Text — (DOCX) [file pgph.0004674.s001.docx]

**S1_Text**

**Predicting and Improving Diagnosis of Tuberculosis Outcomes in South Africa Using Machine Learning Approaches**

***Random Forests (RFs) and Decision Trees (DTs)***

Random forest and Decision trees remain popular machine learning methods for multiple non-parametric regression and classification tasks. Due to their understandability and intuitive decision-making diagrams, these algorithms are the preferred choice for medical scientists when performing disease diagnosis tasks. Since DTs are based on a single tree, their generalizability is limited, thereby motivating the development of RF. RF is an extension of DTs where subsets of training data and randomly selected predictors are used to build multiple trees. The final model is an aggregate of various trees. Due to this stochastic process, it is robust enough to overfit, which is a key challenge in ML. Like DT, RF relies on the recursive splitting process for a classified outcome. It starts at a root node and, using a set of predictors and decision rules, recursively splits the data until an end node is reached. The decision to split relies on the information gain (IG) criterion, which determines whether a particular predictor and the rules it produces yield a higher IG. Through this, the contribution of each predictor to the model can also be calculated and ranked. In our study, we used the IF metric to estimate the importance of variables. We split our data into training and test sets based on the 70:30 rule, which is common in ML tasks. Our data was unbalanced; almost 4 percent had TB, and 96 percent did not. To avoid the impact of the imbalanced structure on our model performance and robustness, we combined stratified sampling with the down-sampling approach. Although some researchers have utilized the Synthetic Minority Over-sampling Technique (SMOTE) in the R environment, we employed a customized approach to sample the majority class down to achieve a 1:3 ratio, which provided a good balance of performance metrics. We used a grid search approach for parameter tuning to obtain a combination of parameters (number of trees, tree depth, and number of trees) that achieves high accuracy. For example, two features and 500 trees were chosen as deciding parameters that achieved high accuracy and a kappa coefficient in the model search space. A five-fold cross-validation approach was used. Specifically, we divided the training data into five equal-sized folds. For every iteration step, we used four subsets to train the model, and the remaining subset was used for validation. We repeated this process five times to ensure that each subgroup served as the validation set once. The accuracy of the metric used to choose the ideal hyperparameter configuration was calculated as the average of the validation accuracy over the five cross-validation subsets. This ensured that the selection accounted for variance across different data subsets.

***Artificial Neural Networks (ANN)***

An artificial neural network is a type of machine learning model, specifically a multi-layer perceptron. This robust pattern recognition algorithm has also played a significant role in regression and classification in health research. The architecture includes computational units, neurons, weighted links that connect neurons, and activation functions. ANNs technically involve three layers: the input layer, which receives inputs; the hidden layer, where computational processes occur; and the output layer, where results are generated. Our study used the sigmoid activation function. We used size (the number of neurons in the hidden layer), decay, and the number of hidden layers as parameters to tune our model. Specifically, we tuned the model using four values (5, 10, 15, and 20) for the number of neurons in the hidden layer. Similarly, we used four scalars (0.1, 0.01, 0.001, and 0.0001) for the regularization parameter. This model was fitted using the Caret R package, where iterative-based optimization was employed to determine the optimal decay and size parameters that achieved the desired result, based on the average accuracy and kappa coefficients. The desired parameters for our model were 1e-04 for the decay and 20 for the size/number of neurons in the hidden layer based on the five-fold cross-validation. The number of hidden layers was fixed at one and did not vary during the tuning process, while the rest of the parameters (decay and number of neurons) were allowed to differ using a grid search approach. We used a mean reduction in variable weights for the variable importance ranking, based on a specific input to the hidden layer, as per the Garson Algorithm. The absolute weights are normalized by default into a 0-100% scale. The variable with the highest absolute normalized weight is ranked highest.

***Stochastic Gradient Boosting Machines (SGBMs)***

Stochastic Gradient Boosting Machines is a boosting machine algorithm, an ensemble-learning technique that combines weak decision trees sequentially to build a robust model. It is currently considered one of the most rigorous and accurate ML models, competing with deep learning. The model begins by fitting a weak learner— a shallow decision tree with a limited number of nodes and branches. The first weak learner's error/residual (the difference between the predicted and observed values) is used to fit the subsequent model. This process repeats in an iterative sequential order. In this case, current models correct the error of the previous ones, thus reducing overall model residuals. This process is better described as a boosting process. This sequential process happens stochastically, where a random subset of predictors and data points is used to fit individual weak learners. This randomness, similar to RF, helps mitigate the risk of overfitting. The model is optimized using the gradient descent approach, where the algorithm follows the direction of steepest error gradient decay. Hyperparameters, including the learning rate, which controls the contribution of each tree to the final model, and regularization, which controls overfitting issues (e.g., tree depth and number of samples to split a node), are used to tune the model for better generalization. We used five-fold cross-validation for model evaluation; the training data is randomly divided into five equal-sized subsets, where the model is fitted iteratively. A grid-based search optimization method was used to select the most accurate parameter combination, ultimately providing the final model. The absolute values used for the model were number of trees = 150, interaction depth = 3, shrinkage = 0.1, and number of observations used to create a terminal node = 10. The shrinkage and terminal node observation parameters were constant for the model fitting process. The variable importance was computed based on two criteria: (1) the number of times a particular variable was used in splitting across all the trees, and (2) how much the variable could help reduce the log loss through the tree splitting process. These two are weighted, summed, and then normalized on a scale of 0 to 100.

***Support Vector Machines (SVMs)***

SVM is a non-probabilistic classifier that has garnered researchers’ attention due to its reliable performance in land cover classification, text classification, and disease prediction. The SVM algorithm projects the data points into a high-dimensional feature space where a hyperplane discriminates between points of two classes. The objective function aims to maximize the margin between the two closest points belonging to different classes. With a complex pattern, linear plane separators may fail to classify correctly. Therefore, kernel functions, such as polynomials and radial-based functions, handle non-linear patterns/trends. In SVM, a five-fold cross-validation was also employed, along with a grid-based search for hyperparameter tuning. Since Caret did not have a built-in SVM algorithm, we used the e1071 package for this task. A radial function, with a cost of 1 and support vectors of 11752, was used to fit the final model. A five-fold cross-validation approach was used. Since we used a radial based on the model training, the direct use of the feature weights would be inappropriate. As a result, we employed a recursive feature elimination (RFE) approach to calculate variable importance. The default RFE setting was used to select the metric to remove the recursive feature.

**Logistic Regression**

We used a logistic regression, generalized linear model with a binomial link function. It models the log-odds of TB as a linear function of the predictors. We fitted this model using the caret package and 5-fold cross-validation. We fine-tuned the model using generalized logistic regression from the glmnet package, focusing on the regularization terms. Using the grid-based search optimization, we experimented with alpha values (0, 0.5, and 1) and ten lambda values, logarithmically spaced between 10^14 and 10^1. Through the five-fold cross-validation, the alpha value was determined to be zero, indicating that the Ridge Logistic Regression with L2 regularization was used. Additionally, a lambda value of approximately 0.0166 was determined. These final hyperparameter sets were used for the final model, as they produced the highest AUC-ROC across all the validation folds.
