# Supplementary material for: Predicting and improving diagnosis of tuberculosis outcomes in South Africa using machine learning techniques
Source: PLOS Glob Public Health. 2025 Nov 7;5(11):e0004674. doi: 10.1371/journal.pgph.0004674 (PMC12594327; doi:10.1371/journal.pgph.0004674)
Supplement: Attachment — Submitted filename: PGPH-D-24-02823_reviewer_EE_12-29-2024.pdf [file pgph.0004674.s002.pdf]

PLOS Global Public Health

Predicting Tuberculosis (TB) with Machine Learning Approaches: An Effort to Improve Tuberculosis Diagnosis in South Africa

--Manuscript Draft--

**Manuscript Number:**	PGPH-D-24-02823
**Article Type:**	Research Article
**Full Title:**	Predicting Tuberculosis (TB) with Machine Learning Approaches: An Effort to Improve Tuberculosis Diagnosis in South Africa
**Short Title:**	Predicting Tuberculosis with Machine Learning
**Corresponding Author:**	Razak M Gyasi, PhD, PD<br>African Population and Health Research Center<br>Nairobi, Nairobi KENYA
**Order of Authors:**	Moses Asori
	Desmond Mbe-Nyire Mpuure
	Daniel Katey
	Razak M. Gyasi
**Keywords:**	machine learning; Tuberculosis; random forest; gradient boosting machines; support vector machines
**Abstract:**	Background: Despite the South Africa Ministry of Health and Social Welfare's efforts to combat tuberculosis (TB) through the "National Strategic Plan (NSP) for addressing HIV, STIs, and TB," progress in preventive and eradication efforts has been hindered by reliance on ineffective diagnostic methods.<br>Methods: Data from the National Income Dynamics Survey, conducted by the Southern African Labour and Development Research Units, were utilized. The dataset underwent a 70:30 train-test split for Random Forest (RF), Decision Trees (DTs), Support Vector Machines (SVMs), Gradient Boosting Machines (SGBMs), and Artificial Neural Networks (ANNs). Hyperparameter tuning and impurity measures were employed for variable-importance rankings.<br>Results: RF achieved 89.02% accuracy, 87.50% sensitivity, and 90.54% specificity. DT achieved 89.21% accuracy, 90.92% sensitivity, and 87.49% specificity. ANN yielded 77.91% accuracy, 81.72% sensitivity, and 74.07% specificity. SGBMs demonstrated 89.09% accuracy, 91.32% sensitivity, and 86.84% specificity. SVMs showed 86.88% accuracy, 90.03% sensitivity, and 83.70% specificity.<br>Conclusion: ML techniques, with accuracy rates mostly surpassing 80%, offer promise in enhancing TB prediction and diagnosis, especially in resource-constrained settings like Sub-Saharan Africa.
**Additional Information:**	
**Question**	**Response**
**Financial Disclosure**<br><br>Enter a financial disclosure statement that describes the sources of funding for the work included in this submission and the role the funder(s) played. This includes grants and any commercial funding of the work or authors.	This research received no specific grant from any funding agency, commercial or not-for-profit sectors.

Powered by Editorial Manager® and ProduXion Manager® from Aries Systems Corporation

This statement will be typeset if the manuscript is accepted for publication.<br><br>*Please review the submission guidelines and the instructions link below for detailed requirements and guidance.*	
**Competing Interests**<br><br>On behalf of all authors, disclose any competing interests that could be perceived to bias this work.<br><br>This statement will be typeset if the manuscript is accepted for publication.<br><br>*Please review the instructions link below and PLOS Global Public Health's competing interests policy to determine what information must be disclosed at submission.*	The authors declared that no direct or indirect competing interests exist.
**Data Availability**<br><br>Before publication, Authors are required to make fully available and without restriction all data underlying their findings. Please see our PLOS Data Policy page for detailed information on this policy.<br><br>A **Data Availability Statement**, detailing where the data can be accessed, is required at first submission. Insert your Data Availability Statement in the box below.<br><br>Please see the data reporting section of our submission guidelines for instructions on what you need to include in your Data Availability Statement.<br><br>This statement will be typeset if the manuscript is accepted for publication.	Data for the analysis is available with corresponding author upon a reasonable request

PLOS allows rare exemptions to address legal and ethical concerns. If you have legal or ethical restrictions, please use the box below to detail these in full sentences for the Journal team to consider.	

Powered by Editorial Manager® and ProduXion Manager® from Aries Systems Corporation

Manuscript
Click here to access/download;Manuscript;Main manuscript_11_17_2024.docx

Predicting Tuberculosis (TB) with Machine Learning Approaches: An Effort to Improve Tuberculosis Diagnosis in South Africa

Moses Asori[1,2], Desmond Mbe-Nyire Mpuure[3], Daniel Katey[4*], Razak M. Gyasi*[5]

[1]Department of Earth, Environmental and Geographical Sciences, University of North Carolina, Charlotte, 28223, USA

[2]Center for Applied Geographic Information Science, [1]Department of Earth, Environmental and Geographical Sciences, University of North Carolina, Charlotte, 28223, USA.

[3]Department of Fundamentals of Economic Analysis, University of Alicante, Sant Vicent del Raspeig, Spain.

[4]Trent Centre for Aging & Society, Trent University, Peterborough, Ontario, Canada

[5]African Population and Health Research Center, Nairobi, Kenya

[*]Corresponding Author

Razak M. Gyasi[5]

[5]African Population and Health Research Center, Nairobi, Kenya

Email: rgyasi.research@gmail.com.

Abstract

Background: Despite the South Africa Ministry of Health and Social Welfare's efforts to combat tuberculosis (TB) through the "National Strategic Plan (NSP) for addressing HIV, STIs, and TB," progress in preventive and eradication efforts has been hindered by reliance on ineffective diagnostic methods.

Methods: Data from the National Income Dynamics Survey, conducted by the Southern African Labour and Development Research Units, were utilized. The dataset underwent a 70:30 train-test split for Random Forest (RF), Decision Trees (DTs), Support Vector Machines (SVMs), Gradient Boosting Machines (SGBMs), and Artificial Neural Networks (ANNs). Hyperparameter tuning and impurity measures were employed for variable-importance rankings.

Results: RF achieved 89.02% accuracy, 87.50% sensitivity, and 90.54% specificity. DT achieved 89.21% accuracy, 90.92% sensitivity, and 87.49% specificity. ANN yielded 77.91% accuracy, 81.72% sensitivity, and 74.07% specificity. SGBMs demonstrated 89.09% accuracy, 91.32% sensitivity, and 86.84% specificity. SVMs showed 86.88% accuracy, 90.03% sensitivity, and 83.70% specificity.

Conclusion: ML techniques, with accuracy rates mostly surpassing 80%, offer promise in enhancing TB prediction and diagnosis, especially in resource-constrained settings like Sub-Saharan Africa.

Keywords: machine learning; tuberculosis; random forest; gradient boosting machines; support vector machines

Introduction

Tuberculosis (TB) remains a significant global health challenge, with over 10 million new cases reported annually and approximately 1.7 million recorded deaths in 2022 (1). Despite substantial progress in diagnosis and treatment, the complex dynamics of TB transmission and the variability in clinical manifestations pose significant obstacles to timely and accurate detection (2,3). This challenge is particularly pronounced in the Southeast Asian and African regions, where factors such as co-infections, drug resistance, and social determinants of health compound the complexity (1), necessitating innovative approaches for effective disease control. In regions grappling with a high TB burden, like South Africa, the impact extends beyond individual health, permeating communities and straining healthcare systems (2–4). Thus, achieving the global targets outlined in the *End TB Strategy* mandates improved treatment and preventive measures and a transformative shift in the approach to TB diagnosis (1).

On the other hand, recent advancements in machine learning applications within the scope of TB diagnosis and treatment appear to offer a promising avenue for addressing these challenges. Studies, including the work of Yadav et al. (5), showcase the enhanced accuracy of deep learning in classifying X-ray images of potential TB patients, facilitating early detection and subtype differentiation. Furthermore, Sharma et al. (6) explored TB drug resistance profiles using machine learning, guiding targeted treatment strategies. Additionally, Acharya et al. (7) employed a progressive resizing approach to train models for automatic TB diagnosis, integrating fine-tuned Normalisation-Free Networks (NFNets) and the Score-Cam algorithm to highlight specific regions in chest X-rays for detailed inference. This scholarly evidence underscores the practical utilization of machine learning and Artificial Intelligence (AI) in real-world clinical contexts. As we navigate the evolving landscape of TB diagnosis, it becomes

evident that machine learning stands at the forefront, offering innovative solutions and tangible advancements in our fight against this age-long global health threat.

The nation of South Africa remains a big name in contemporary TB-related reports and literature globally (2,4,8–10). In 2020, WHO updated the list of the top 30 TB-burdened countries globally, including South Africa (11). Also, among these unique countries, it was discovered that South Africa had the third-highest absolute count of TB-reported cases, 172,200, and the fifth-highest prevalence of TB patients, 304,000 (11). The country also assumed a leading role by having the highest number of TB cases concurrent with HIV, reaching 81,800, and appeared as the second-ranking country in the diagnosis of multidrug-resistant (MDR)-TB cases, with a significant count of 7,100 (12). Although South Africa witnessed some reductions in TB cases in 2023 (1), it appears that South Africa still bears a substantial share of the global TB burden, necessitating innovative approaches to effectively combat and, if possible, eradicate the disease in the country. A recent study by Osman et al. (3), which delved into the systemic drivers of TB-related mortality in the country, provides some valuable insights into the complex interplay of some health systems factors and how they contribute to the disease prevalence in the country.

However, a critical gap in their analysis (probably due to their methodological approach) lies in exploring innovative technological interventions for improving TB diagnosis in the country. It is crucial to fill this critical knowledge gap for several reasons: (1) to enhance the accuracy of detecting and identifying TB cases, (2) to ensure timely initiation of treatment for affected individuals, and (3) to advance research and development in the fight against TB. Therefore, building upon the successes of previous studies that utilized machine-learning approaches and techniques to study TB and related diseases, our research aims to propose some

machine-learning methodologies for predicting and improving TB diagnosis outcomes in South Africa. Fundamentally, our study seeks to bridge the gap between understanding the systemic drivers of TB mortality and implementing cutting-edge interventions that directly impact the diagnostic landscape and proffer some plausible recommendations to achieve global targets and milestones for reductions in TB incidence and diseases by 2030 (2).

Materials and Methods

Data and Sampling

We used data from the National Income Dynamics Survey (NIDS), a comprehensive survey conducted by the Southern African Labour and Development Research Units (SALDRU) in South Africa (13). The survey employed a two-stage cluster sample design to sample households during the base wave. In the first stage, a sample of 400 primary sampling units (PSUs) was selected from the Master sample of 3000 PSUs used by Statistics South Africa for its Labour Force Survey and General Household Survey (LFSGHS) between 2004 and 2007. Notably, the NIDS PSUs constitute a subset of the Master sample. The 400 PSUs were randomly chosen from the strata of 52 district councils (DCs) (13). NIDS, conducted every two years from 2008 to 2017, serves as a nationwide panel survey, offering valuable insights into individual and household characteristics. This longitudinal design enables the tracking of household members, differentiating between continuing sample members (CSM) and new members, such as newborns, included in subsequent waves and referred to as temporary sample members (TSM). This rich dataset facilitates the exploration of dynamic household structures, changes in living conditions, and overall well-being.

Given the burden of diseases in South Africa, including sexually transmitted diseases, hypertension, tuberculosis, fever, stroke, and others, the NIDS is particularly valuable for understanding recent changes in the living conditions of the population and their epidemiologic profiles. We pool data from the last two waves of NIDS for analysis. This approach enables us to provide policy suggestions based on recent trends. Our choice of surveys in the analysis is aligned with the study's objectives, irrespective of whether the individual is a CSM or TSM. The attrition rate in the sample for the NIDS data is estimated to be about 18 percent, with the high-income holders dropping from the survey. The survey started its first round in 2008 with 28,000 residents of 7,303 households. This data has national coverage spanning all nine provinces and 52 districts of South Africa. Our study's final sample is obtained from the last two waves, representing 17,961 in 2014/2015 and 19,267 in 2017, giving a total sample of 37,228. This final sample is obtained after cleaning and considering the missing values of the relevant variables of interest.

Outcome Variable

Our outcome or target variable for training the ML models was TB. It was a binary categorical variable encoded as 0 or 1; 1 indicated those with TB, and 0 represented those without. Despite the significant strides in eliminating TB, it remains a significant epidemiologic burden to the country partly due to a lack of precise diagnostic protocols. South Africa was ranked sixth globally for the high burden of TB (0.4-0.6 million) (14). While several strategic targets by the WHO in 2000 and 2015 have achieved their goal of halving TB (14), in the South, the confluence of TB, HIV/AIDS and increasing cardiometabolic diseases are achieving this target challenging (15). Even though the TB burden has attracted notable attention from the Ministry of Health and Social Welfare through the development of the National Strategic Plan

(NSP) for tackling "HIV, STIs, and TB," precise predictive methods for its diagnosis have received limited research attention in the country and SSA in general. During the survey exercise, respondents were asked whether they had ever been medically diagnosed with TB within the last 30 days of the survey. For example, respondents were asked, "Within the last 30 days, have you been diagnosed with TB?" Those who responded yes were categorized as TB-infected and zero otherwise.

Predictors/features

After the multicollinearity test and feature selection process, sixteen predictors or features were used to train the ML models. They included blood cough, persistent cough, diabetes, diarrhea, high blood pressure, stroke, cancer, age, alcohol consumption, heart problems, fever, cigarette use, severe weight loss, self-rated health condition, and chest pain (See Table 1). All these variables were self-reported. As a country where there is a high confluence of infectious diseases such as HIV/AIDS, TB, and many other chronic non-infectious diseases (CNDs), the health system is stressed, requiring innovative approaches to support resourceful decision-making.

Insert Table 1 here

TB risk is influenced by multiple risk factors, with notable ones being immune-suppressive diseases such as HIV/AIDS. For example, the risk of TB in South Africa has increased proportionally to the rate of increase in HIV/AIDS cases. Immune suppressive diseases can activate latent TB and catalyze its progression. Because HIV/AIDS status was not provided in the database, we used other proxies, including cancer, asthma, blood cough, and persistent cough, as suggestive elements for an immune-compromised individual or those with higher

infectibility tendencies. For example, one clinical study found that a 24-hour cough frequency was statistically significantly linked to TB-positive sputum smear status (p=0.04) (16). While a recent review by Patterson and Wood (17) found that coughing alone is not necessarily a mechanical causal factor in transmission; it is linked to significant infectibility. Therefore, susceptible people, including family members, may easily acquire TB through coughed air droplets (17). Blood cough may also indicate advanced respiratory conditions such as pneumonia, which may suggest an immune-compromised situation (18). These two factors were used as significant indicators for immune-compromised situations.

Also, severe weight loss, blood, persistent sweat/fever, and fatigue are substantial clinical symptoms of active TB (19, 20). Furthermore, an estimated 70 percent of people with diabetes now live in middle and low-income countries, with increasing rates in areas with high TB rates, such as countries in SSA (21). Several reviews and primary studies have demonstrated an association between TB and diabetes. A meta-analysis of 13 studies found a 3-fold increase in TB among people with diabetes as compared to those without diabetes. Evidence suggests that having diabetes disrupts the innate and adaptive immune system, thereby affecting the body's ability to effectively fight the tuberculosis bacterium. Furthermore, several meta-analyses and systematic have empirically demonstrated the relationships between TB and smoking. A systematic review by Bates and colleagues found a relative risk of 2.3–2.7 TB among smokers as compared to non-smokers (22). A similar review by [25] found a 2-fold risk for TB among smokers as compared to non-smokers, suggesting that smoking remains a significant factor for TB development and transmission. Generally, a 2-fold increased risk of TB following alcohol consumption has also been found (23).

Considering that a more significant share of South Africans consume alcohol (24–26) and are smokers, they were deemed crucial to our TB prediction with ML. Age has also been found to be a risk for TB, with increased risk among children and older adults. However, besides increasing the risk, it also moderates acquisition pathways. For example, one study found that children under two years of age acquired TB from home, whereas those over two were acquired from community-level exposure (27). We included age as a feature in our prediction. Even though malnutrition has been shown as another risk factor for TB development, in our study, we used severe weight loss as a proxy for severe nutrition-associated wasting. We also considered high blood pressure based on systolic and diastolic blood pressures. High blood pressure was defined as a systolic pressure of >90mm/hg and a diastolic pressure of 140mm/hg. Even though one systematic review study found no significant link between TB and hypertension, it did caution against the interpretation of the finding, given the limitation of study designs used for the meta-analysis (28). In contrast, one nationwide cohort study found a significant association between TB and hypertension (29). Also, evidence exists for the association between diabetes and TB (30, 31), warranting inclusion for training our models.

Pre-processing

Pre-processing is crucial in ML, as insufficient data may generate bad results regardless of the robustness of the ML model. This study's pre-processing included handling missing data, correcting variable names, observing outliers, and transforming variables where necessary. Summary statistics and charts were used to explore our data's nature and detect anomalies, including missing data, wrongly spelled variables, and wrong data structure. For this study, approximately 0.84 percent of data points were missing. Assuming these data points were missing at random, we used multiple imputations by chained equation (mice) technique in the R

environment to input the missing values based on the random forest technique. A bivariate regression between TB and the predictors was conducted to avoid including unnecessary features for the prediction. All predictors with a p-value >10 percent were excluded. The retained predictors listed above were subjected to the multi-collinearity test using the correlation matrix approach. None of the variables had a correlation coefficient >0.5 in the correlation matrix. Therefore, it was assumed that no multi-collinearity existed that could threaten our model adequacy. All our variables except age were categorical.

Analysis

ML is a non-parametric but robust model for dealing with complex and non-linear patterns within data, allowing it to be more favorable to current data science tasks. It is a cyclical process that may evolve from data acquisition, preprocessing/data engineering/feature engineering, model calibration, and validation. A simplified overview is given in [Figure 1]. We used five ML techniques for our classification task. There is random forest (RF), decision trees (DT), artificial neural networks (ANN), stochastic gradient boosting machines (SGBM), and support vector machines (SVM). We used a grid search approach for tuning the hyperparameters. See supplementary files.

Insert figure 1 here

Random Forests and Decision Trees

Random forest and DTs remain popular ML for multiple non-parametric regression and classification tasks. Owing to their understandability and intuitive decisional diagram, they are the favorite algorithms medical scientists use for disease diagnosis tasks. Since DTs are based on a single tree, their generalizability is limited, thereby motivating the development of RF. RF is an

extension of DTs where subsets of training data and randomly selected predictors are used to build multiple trees. The final model is an aggregate of various trees. Due to this stochastic process, it is robust to overfitting, a key challenge in ML. Like DT, RF relies on the recursive splitting process for a classified outcome. It starts at a root node, and using a set of predictors and decisional rules, it splits the data recursively until the end node is reached. The decision to split relies on information gain (IF) criteria, which decides whether a particular predictor and rules produced a higher IF. Through this, each predictor's contribution to the model can also be calculated and ranked. In our study, we used the IF metric to estimate the importance of variables. We split our data into training and test sets based on the 70:30 rule, common in ML tasks. Our data was unbalanced; almost 4 percent had TB, and 96 percent did not. To avoid the impact of the imbalanced structure on our model performance and robustness, we combined stratified sampling with the Synthetic Minority Over-sampling Technique (SMOTE) in the R environment. We used a default setting for the SMOTE. We used a grid search approach for parameter tuning to get a combination of parameters (number of trees, number of trees, and tree depth) that achieve high accuracy. For example, two features and 500 trees were chosen as deciding parameters that achieved high accuracy and kappa coefficient in the model search space. A five-fold cross-validation approach was used.

Artificial Neural Networks (ANN)

ANN is a multi-layer perceptron ML model, a robust pattern recognition algorithm that has also played a significant role in regression and classification in health research. The architecture includes computational units, neurons, weighted links that connect neurons, and activation functions. ANN technically involves three layers: the input layer that receives inputs, the hidden layer where computational processes occur, and the output layer where results are

generated. Our study used the sigmoid activation function. We used size (number of neurons in hidden layer), decay, and number of hidden layers as parameters to tune our model. This model was fit with the caret R package, where iterative-based optimization was used to find the appropriate decay and size parameters that achieve the desired result based on accuracy and kappa coefficients. The desired parameters for our model are for the 10-end superscript to the minus 4-end superscripts. A five-fold cross-validation approach was also used.

Stochastic Gradient Boosting Machines

SGBM is a boosting machine algorithm, an ensemble-learning technique where weak decision trees are combined sequentially to build a robust model. It is currently considered one of the most rigorous and accurate ML, competing with deep learning. The model starts by fitting a weak learner –a shallow decision tree with limited nodes and branches. The first weak learner's error/residual (difference between predicted and observed) is used to fit the subsequent model. This process repeats in iteratively sequential order. In this case, current models correct the error of the previous ones, thus reducing overall model residuals. This process is better described as a boosting process. This sequential process happens stochastically, where a random subset of predictors and data points are used to fit individual weak learners. This randomness, like in RF, helps overcome the risk of overfitting. The model is optimized using the gradient descent approach, where the algorithm follows the steepest error gradient decay direction. Hyperparameters, including learning rate, which controls the contribution of each tree to the final model, and regularization, which controls overfitting issues (e.g., tree depth and number of samples to split a node), are used to tune the model for better generalization. We used five-fold cross-validation for model evaluation; the training data is randomly divided into five equal-sized subsets, where the model is fitted iteratively. A grid-based search optimization method selected

the most accurate parameter combination to provide the final model. The absolute values used for the model were number of trees = 150, interaction depth = 3, shrinkage = 0.1, and number of observations used to create a terminal node = 10. The shrinkage and terminal node observation parameters were constant for the model fitting process.

Support Vector Machines

SVM is a non-probabilistic classifier that has gained researchers' attention for its reliable performance in land cover classifiers, text classification, and disease predictions. The SVM algorithm projects the data points into a high-dimensional feature space where a hyper-plane discriminates between points of two classes. The objective function seeks to maximize the margins between the two closest points belonging to two separate classes. With a complex pattern, linear plane separators may fail to classify correctly. Therefore, kernel functions like polynomials and radial-based functions handle non-linear patterns/trends. In SVM, a five-fold cross-validation was also used, with grid-based search as hyperparameter tuning. Since Caret did not have a built-in SVM algorithm, we used the e1071 package for this task. A radial function, with a cost of 1 and support vectors of 11752, was used to fit the final model. A five-fold cross-validation approach was used.

Model Performance Assessment

Model validation, such as its generalizability, is crucial to deciding whether or not the model should be relied on. We used a confusion matrix to derive several performance metrics. These metrics assessed model adequacy and reliability. Metrics included accuracy, sensitivity, specificity, F1-score, negative predictive value (NPV) and positive predictive value (PPV). These metrics are calculated as follows;

$$Accuracy = \frac{TP+TN}{TP+TN+FP+FN} \quad [1]$$

$$PPV = \frac{TP}{TP+TN} \quad [2]$$

$$Sensitivity = \frac{TP}{TP+FP} \quad [3]$$

$$Specificity = \frac{TN}{TN+FP} \quad [4]$$

Where TP is the true positive, TN is the true negative, FP is the false positive, and FN is the false negative rate. The area under the curve or receiver operating characteristic (ROC) was used to evaluate the predictive accuracy of the algorithm. It plots iterative TP versus FP values based on various cutoffs. AUC values suggest the ability of the algorithm to discriminate between TB-positive and TB-negative respondents. The difference between no information rate (NIR) and accuracy was used to evaluate the improvement of our models over null models. A *p*-value of <0.05 was used as a significant level.

Results

Summary characteristic

The summary characteristics of our predictor variables are shown in Table 1. A total of 37228 respondents were finally used to train our model. About 3.7 percent reported having been diagnosed with TB, with an average age of 35.09 years (95 percent CI: 34.92-35.26 years). About 21 percent had a fever, and those with persistent cough were 11.1 percent, and blood cough was 1.0 percent. Furthermore, those who reported chest pain were 7.1 percent, cancer was 0.9 percent, and hypertension was 12.0 percent. Also, asthma was 2.3 percent, whereas 1.5 percent reported having heart problems. Also, those who reported having a stroke were 0.7 percent, whereas diabetic respondents were 2.9 percent. Those ever diagnosed with diarrhea

were 3.7 percent, and smoking was 18.1 percent. With alcoholic consumption, those who have never taken alcohol were 31.37 percent, those who rarely drink were 23.23 percent, whereas those who are regular drinkers were 45.39 percent. Concerning self-rated health conditions, 32.39 percent rated their health as excellent, 31.60 percent rated their health as very good, 26.93 percent rated their health as good, 7.05 percent rated it as fair, and 2.03 percent rated their health as poor.

ML prediction results

Based on the confusion matrix, performance characteristics for RF were: accuracy was 89.02 percent (95 percent CI: 88.42, 89.59 percent), sensitivity was 87.50 percent, specificity was 90.54 percent, PPV was 90.32 percent, whereas NPV was 87.79 percent. We also found that the accuracy hypothesis of >NIR had a p-value <0.0001. Concerning DT, accuracy was 89.21 percent (95 percent CI: 88.62, 89.79 percent), sensitivity was 90.92 percent, specificity was 87.49 percent, PPV was 87.99 percent, whereas NPV was 90.53 percent. Also, accuracy > NIR had a p-value <0.0001. Concerning ANN, the accuracy was 77.91 percent (95 percent CI: 77.12, 78.68 percent), sensitivity was 81.72 percent, specificity was 74.07 percent, PPV was 76.05 percent, and NPV was 80.08 percent. Accuracy > NIR had a p-value <0.0001. Also, according to SGBM, the accuracy was 89.09 percent (95 percent CI: 88.49, 89.66 percent), sensitivity was 91.32 percent, specificity was 86.84 percent, PPV was 87.49 percent, whereas NPV was 90.84 percent. Lastly, the results of the SVM are as follows: accuracy was 86.88 percent (95 percent CI: 86.24, 87.5 percent), sensitivity was 90.03 percent, specificity was 83.70 percent, PPV was 84.77 percent, whereas NPV was 89.28 percent (See Table 2 and Table 3).

Insert Table 2 here

Insert Table 3 here

Insert Figure 2 here

Blood cough consistently came top in all the ensemble models for variable importance. For example, in the RF model, blood cough was ranked first, followed by stroke, severe weight loss, chest pain, and persistent cough (See Figure 3). The last three variables that contributed least to the RF were hypertension, age, and cancer.

Insert Figure 3 here

In DT, the top five most essential predictors in decreasing order of significance were heart problems, asthma, severe weight loss, stroke, and blood cough. However, the last three variables contributing the least were cancer, age, and alcohol. In an ANN model, the top five most essential variables in decreasing order of importance were blood cough, stroke, asthma, severe weight loss, and hypertension. On the contrary, the three least critical variables were cancer, age, and self-rated health status. For SGBM, the top five most essential predictors in decreasing order of importance were blood cough, stroke, severe weight loss, asthma, and chest pain. The last three least important ones were hypertension, cancer, and alcohol (See Figure 3).

Discussion

Tuberculosis remains a critical health challenge in developing countries, alongside HIV/AIDS and other non-communicable diseases (NCDs). In South Africa, the burden of TB on

healthcare systems is substantial (15). Even though the TB burden has attracted notable attention from the South Africa Ministry of Health and Social Welfare through the development of the National Strategic Plan (NSP) for tackling “HIV, STIs, and TB (32), preventive and eradication efforts have been stalled, partly due to outdated and inefficient diagnostic and predictive methods. Therefore, the cruciality of our current study cannot be underestimated, especially in less resource settings where the clinical diagnosis of TB and underlying risk factor identification remains primarily constrained. Using nationally representative data, five established machine learning models were used to determine the most precise model for enhancing TB diagnosis and prediction in clinical practice. Besides the merit of individual-level intervention, our study can shape system-based thinking regarding program designs and resource allocation (33). Also, the process of facilitating corrective actions through reforming existing patient-oriented decisional frameworks may be enhanced. In this study, we have utilized sixteen non-invasive personal information, including self-reported blood cough, persistent cough, diabetes, diarrhea, high blood pressure, stroke, cancer, age, alcohol consumption, heart problems, fever, cigarette use, severe weight loss, self-rated health condition, and chest pain to discriminate TB status precisely among participants.

RF, DTs, SGBMs, ANN, and SVMs were used for the predictive exercise. We found substantive accuracies across all models, with each accuracy exceeding 70 percent. We also found at least ROC above 84 percent across all models (See Figure 2). However, RF, DTs, and SGBMs, which comprised ensemble learners, outperformed all the other models with accuracies of 89.02 percent, 89.21 percent, and 89.09 percent, respectively. They were, on average, 2.2 percent higher in accuracy than SVMs (86.88 percent) and 12 percent higher than ANNs (~77 percent). Using an algorithmic process to discriminate clinical outcomes has several advantages

over traditional statistics, such as (1) reducing the risk of functional misspecifications, (2) enhancing the handling of non-linearity structure within data, and (3) improving the ability to test for model generalization through model validation, which is challenging to achieve adequately in traditional statistical approaches. Unsurprisingly, AI and ML approaches are increasingly used to inform clinical and epidemiological practice. For example, studies, including the work of (5), showcase the enhanced accuracy of deep learning in classifying X-ray images of potential TB patients, facilitating early detection and subtype differentiation. Furthermore, (6) explored TB drug resistance profiles using machine learning, guiding targeted treatment strategies. Additionally, (7) employed a progressive resizing approach to train models for automatic TB diagnosis, integrating fine-tuned Normalisation-Free Networks (NFNets) and the Score-Cam algorithm to highlight specific regions in chest X-rays for detailed inference.

Besides high accuracy and sensitivity, there was also a perfect balance between sensitivity and specificity, demonstrating the clinical relevance of our study. Despite DTs exhibiting comparative accuracy as SGBMs, the latter demonstrated superior sensitivity and specificity results, indicating the robustness of SGBMs. Of course, SGBMs are one of the most robust models presently, sometimes outperforming deep learning models; it is, therefore, not surprising that it will have such superiority over the single-tree decision model, which is prone to overfitting. Besides predictive outcomes, understanding variables' contribution to model performance provides crucial insights for developing preventive strategies. For instance, identifying the most influential predictors for TB enables clinicians to target the most vulnerable epidemiological and demographic groups, facilitating more effective resource allocation. Across all the ensemble models (RF, SGBMs, and DTs) and ANNs, persistent cough, blood cough, and severe weight loss appeared very influential in the discrimination of TB status among our

participants. This finding corroborates with other studies. For example, one clinical study found that a 24-hour cough frequency was statistically significantly linked to TB-positive sputum smear status (p=0.04) (16). While a recent review by Patterson and Wood (17) found that coughing alone is not necessarily a mechanical causal factor in transmission, it is linked to significant infectibility. Blood cough may also indicate an advanced level of pathogenic invasion and immunosuppression. Also, severe weight loss, blood, persistent sweat/fever, and fatigue are substantial clinical symptoms of active TB (19, 20). Therefore, it is unsurprising that severe weight loss and persistent cough were also significant features in our predictive models. Additionally, all models found stroke and asthma as influential factors.

Even though differences in algorithmic pattern recognition and model complexity may lead to different variable importance rankings, the consistency of stroke as the second most influential predictor in most of our models suggests its criticality in the clinical identification of TB. A complex-mediated immunosuppression following stroke may increase the risk of dysphagia and aspiration, which may elevate the risk of respiratory diseases such as TB. However, we need to emphasize that the linkage between TB and stroke is not straightforward: it is multifaceted and highly mediated by many other underlying health conditions, such as diabetes and hypertension. Therefore, causal interpretation in our study should be made with caution. We also found that chest pain and fever contributed moderately to our prediction across almost all models. It is, therefore, unstartling that the presence and severity of fever have been used to evaluate anti-TB drug effectiveness in some cases. One crucial finding was that, despite the grid-search optimization technique used, all the ensemble models outperformed ANNs and SVMs. This may be explained by the presence of non-linearity in our data structure. For example, SVMs and ANN models may be less efficient in mathematically mapping predictors to

the response variable(s) in non-linear patterns. Additionally, ensemble models like RFs and DTs are robust to noisy data, outliers, and small sample effects. Therefore, ensemble decision-based models may be crucial to overcoming predictive challenges, especially in financial and data-constrained geographies such as South Africa and SSA. Generally, our study demonstrates the potential of MLs in enhancing clinical assessment, identification, and prediction of TB in Africa, where its prevalence is still relatively high.

Limitations

The study relied on cross-sectional and self-reported data, potentially introducing recall biases and limitations in capturing the temporal aspects of disease conditions. However, by merging data from a two-period survey, we aimed to enhance the predictive power of our models. It is essential to acknowledge that machine learning models can perpetuate demographic biases in their prediction and algorithmic decisions, which may have significant implications for patient care and health decision-making processes. Nevertheless, due to data constraints, we were unable to train and validate our models across racial groups, particularly for under-represented groups such as Whites and Asians, comprising less than 20 percent of South Africa's racial composition. Therefore, we cannot be conclusive about the predictive nature of our models for these under-represented groups. Additionally, gender-specific training and testing were not conducted. Nevertheless, given the roughly equal gender distribution (45:55 ratio) in our dataset, we anticipate that the predictive accuracies and diagnostic guidance derived from our study can be reasonably applied to both males and females with minimal demographic biases. Lastly, we focused on general TB status due to data constraints. Therefore, a sub-group assessment on extra-pulmonary TB, which comprises 15 percent of TB cases in South Africa, was not conducted. Therefore, extrapolation to specific types of TB should be done with caution.

Conclusion

TB continues to present ravaging effects on developing nations, and South Africa is no exception. To contribute to enhancing clinical practice through the prediction and diagnosis of TB, we developed five known ML models: RF, SVM, SGBMs, DTs, and ANN, and compared their efficiencies. We used sixteen non-intrusive personal information for the prediction. We found that tree-based models (SGBMs, DTs, and RFs) outperformed ANN and SVMs. However, based on accuracy, all models achieved an accuracy of >70 percent, with SGBMs and RFs achieving satisfactory sensitivity, specificity, ROCs, and accuracy measures. Our study may be crucial to TB prevention and management in the immediate and long term.

Declarations

- **Consent for publication**

All authors approve of the manuscript to be published

- **Availability of data and materials**

Data for the analysis is available with corresponding author upon a reasonable request

- **Competing interests**

The authors declared that no direct or indirect competing interests exist.

- **Authors' contributions**

MA contributed to the study conceptualization and design, did formal statistical analysis, supervised the data collection, interpreted the results, and wrote the initial draft of the paper. DMNM contributed to the study conceptualization and design, supervised the data collection, interpreted the results, and assisted in writing the manuscript. DK contributed to writing and editing the paper and provided supervision in data collection. RMJ contributed to

conceptualization, supervised the writing of the paper, and edited the final write-up. All authors read and approved the final draft of the manuscript

- **Conflict of Interest**

The authors declare none.

- **Funding details:**

This research received no specific grant from any funding agency, commercial or not-for-profit sectors.

Reference

1. World Health Organization (WHO). Tuberculosis [Internet]. 2023 [cited 2024 Jan 16]. Available from: https://www.who.int/news-room/fact-sheets/detail/tuberculosis
2. Foster N, Vassall A, Cleary S, Cunnama L, Churchyard G, Sinanovic E. The economic burden of TB diagnosis and treatment in South Africa. Soc Sci Med. 2015 Apr 1;130:42–50.
3. Osman M, Karat AS, Khan M, Meehan SA, von Delft A, Brey Z, et al. Health system determinants of tuberculosis mortality in South Africa: a causal loop model. BMC Health Serv Res. 2021 Dec 1;21(1):1–11.
4. Padayatchi N, Daftary A, Naidu N, Naidoo K, Pai M. Tuberculosis: Treatment failure, or failure to treat? Lessons from India and South Africa. BMJ Glob Health. 2019 Jan 1;4(1).
5. Yadav O, Passi K, Jain CK. Using Deep Learning to Classify X-ray Images of Potential Tuberculosis Patients. In: Proceedings - 2018 IEEE International Conference on

Bioinformatics and Biomedicine, BIBM 2018. Institute of Electrical and Electronics Engineers Inc.; 2019. p. 2368–75.

6. Sharma A, Machado E, Lima KVB, Suffys PN, Conceição EC. Tuberculosis drug resistance profiling based on machine learning: A literature review. Brazilian Journal of Infectious Diseases. 2022 Apr 1;26(1):102332.

7. Acharya V, Dhiman G, Prakasha K, Bahadur P, Choraria A, Sushobhitha M, et al. AI-Assisted Tuberculosis Detection and Classification from Chest X-Rays Using a Deep Learning Normalization-Free Network Model. Comput Intell Neurosci. 2022;2022.

8. Oni T, Youngblood E, Boulle A, McGrath N, Wilkinson RJ, Levitt NS. Patterns of HIV, TB, and non-communicable disease multi-morbidity in peri-urban South Africa- a cross sectional study. BMC Infect Dis. 2015 Jan 17;15(1).

9. Kigozi G, Heunis C, Chikobvu P, Botha S, van Rensburg D. Factors influencing treatment default among tuberculosis patients in a high burden province of South Africa. International Journal of Infectious Diseases. 2017 Jan 1;54:95–102.

10. Heunis J, Kigozi N, Chikobvu P, health SBB public, 2017 undefined. Risk factors for mortality in TB patients: a 10-year electronic record review in a South African province. Springer. 2017 Jan 6;17(1).

11. Tomlinson C. In-depth: What new WHO TB numbers mean for SA • Spotlight [Internet]. 2023 [cited 2024 Jan 18]. Available from: https://www.spotlightnsp.co.za/2023/11/10/in-depth-what-new-who-tb-numbers-mean-for-sa/

12. Scott LE, Shapiro AN, Da Silva MP, Tsoka J, Jacobson KR, Emch M, et al. Integrating Molecular Diagnostics and GIS Mapping: A Multidisciplinary Approach to Understanding Tuberculosis Disease Dynamics in South Africa Using Xpert MTB/RIF. Diagnostics. 2023;13(20):1–15.

13. University of Cape Town. National Income Dynamics Survey (NIDS). 2021. National Income Dynamics Survey (NIDS).

14. WHO. Global Tuberculosis Control. 2013. Global Tuberculosis Control.

15. Knight GM, Dodd PJ, Grant AD, Fielding KL, Churchyard GJ, White RG. Tuberculosis prevention in South Africa. PLoS One. 2015 Apr 7;10(4).

16. Turner RD, Birring SS, Darmalingam M, Hooper RL, Kunst H, Matos S, et al. Daily cough frequency in tuberculosis and association with household infection. International Journal of Tuberculosis and Lung Disease. 2018 Aug 1;22(8):863–70.

17. Patterson B, Wood R. Is cough really necessary for TB transmission? Vol. 117, Tuberculosis. Churchill Livingstone; 2019. p. 31–5.

18. Mani CS. Acute pneumonia and its complications. Principles and practice of pediatric infectious diseases. 2018;238.

19. Mandieka E, Saleh D, Chokshi AK, Rivera AS, Feinstein MJ. Latent tuberculosis infection and elevated incidence of hypertension. Vol. 9, Journal of the American Heart Association. American Heart Association Inc.; 2020.

20. Luies L, Preez I du. The echo of pulmonary tuberculosis: Mechanisms of clinical symptoms and other disease-induced systemic complications. Vol. 33, Clinical Microbiology Reviews. American Society for Microbiology; 2020. p. 1–19.

21. Dunachie S, Chamnan P. The double burden of diabetes and global infection in low and middle-income countries. Vol. 113, Transactions of the Royal Society of Tropical Medicine and Hygiene. Oxford University Press; 2019. p. 56–64.

22. Bates MN, Khalakdina A, Pai M, Chang L, Lessa F, Smith KR. Risk of Tuberculosis From Exposure to Tobacco Smoke A Systematic Review and Meta-analysis. Arch Intern Med. 2007;167(4):335–42.

23. Lin HH, Ezzati M, Murray M. Tobacco smoke, indoor air pollution and tuberculosis: A systematic review and meta-analysis. Vol. 4, PLoS Medicine. 2007. p. 0173–89.

24. Necho M, Belete A, Getachew Y. The prevalence and factors associated with alcohol use disorder among people living with HIV/AIDS in Africa: A systematic review and meta-analysis. Subst Abuse Treat Prev Policy. 2020 Aug 24;15(1).

25. Necho M, Belete A, Getachew Y. The prevalence and factors associated with alcohol use disorder among people living with HIV/AIDS in Africa: A systematic review and meta-analysis. Subst Abuse Treat Prev Policy. 2020 Aug 24;15(1).

26. Soepnel LM, Kolkenbeck-Ruh A, Crouch SH, Draper CE, Ware LJ, Lye SJ, et al. Prevalence and socio-structural determinants of tobacco exposure in young women: Data from the Healthy Trajectories Initiative (HeLTI) study in urban Soweto, South Africa. Drug Alcohol Depend. 2022 Mar 1;232.

27. Marais BJ, Gie RP, Schaaf HS, Hesseling AC, Obihara CC, Nelson LJ, et al. The clinical epidemiology of childhood pulmonary tuberculosis: a critical review of literature from the pre-chemotherapy era [State of the Art]. The International Journal of Tuberculosis and Lung Disease. 2004;8(3):278–85.

28. Seegert AB, Rudolf F, Wejse C, Neupane D. Tuberculosis and hypertension—a systematic review of the literature. Vol. 56, International Journal of Infectious Diseases. Elsevier B.V.; 2017. p. 54–61.

29. Chung WS, Lin CL, Hung CT, Chu YH, Sung FC, Kao CH, et al. Tuberculosis increases the subsequent risk of acute coronary syndrome: a nationwide population-based cohort study. The International journal of tuberculosis and lung disease. 2014;18(1):79–83.

30. Kim SJ, Hong YP, Lew WJ, Yang SC, Lee EG. Incidence of pulmonary tuberculosis among diabetics. Tubercle and lung disease. 1995;76(6):529–33.

31. Alisjahbana B, van Crevel R, Sahiratmadja E, den Heijer M, Maya A, Istriana E, et al. Diabetes mellitus is strongly associated with tuberculosis in Indonesia. The international journal of tuberculosis and lung disease. 2006;10(6):696–700.

32. Ministry-of-Health-and-Social Welfare. National Strategic Plan for HIV | TB | STIs. 2024.

33. AlKaabi LA, Ahmed LS, Al Attiyah MF, Abdel-Rahman ME. Predicting hypertension using machine learning: Findings from Qatar Biobank Study. PLoS One. 2020 Oct 1;15(10 October 2020).

Table 1: Predictor variables or features and descriptive statistics

Variable	Observation	Proportion (%)
Fever		

0	29,492	79.1
1	7,736	20.9
Tuberculosis		
0	35,847	96.3
1	1,381	3.7
Persistent cough		
0	33,112	88.9
1	4,116	11.1
Cough with Blood		
0	36,855	99.0
1	373	1.0
Chest pain		
0	34,582	92.9
1	2,646	7.1
Cancer		
0	36,767	99.1
1	323	0.9
Hypertension		
0	32,760	88.0
1	4,468	12.0
Asthma		
0	36,357	97.7
1	871	2.3

Heart problem		
0	36,681	98.5
1	547	1.5
Stroke		
0	36,977	99.3
1	251	0.7
Diabetes		
0	35,860	97.2
1	1,052	2.9
Diarrhea		
0	35,855	96.3
1	1,373	3.7
Smoke		
0	30,471	81.9
1	6,757	18.1
Severe weight loss		
0	36,122	97.0
1	1,106	3.0
Alcohol consumption		
Never	11,680	31.37
Rare	8,649	23.23
Drink	16,899	45.39

Self-rated health		
Excellent	12,059	32.39
Very good	11,763	31.60
Good	10,026	26.93
Fair	2,624	7.05
Poor	756	2.03
Age	37,228	35

Table 2: The confusion Matrix for various ML models

**Random Forest**			
		Reference	
Predictions		0	1
	0	4887	524
	1	698	5017
**Decision Trees**			
	0	5078	693
	1	507	4848
**Artificial Neural Network**			
	0	4564	1437
	1	1021	4104
**Stochastic Gradient Boosting Machines**			
	0	5100	729
	1	485	4812
**Support Vector Machine**			

	0	5028	903
	1	557	4638

Table 3: The performance metrics for various ML models

	Random Forest	Decision Trees	Artificial Neural Network	Stochastic Gradient Boosting Machines	Support Vector Machine
**Metric Name**	Metric value				
**Accuracy**	0.8902 [95% CI: 0.8842, 0.8959]	0.8921[0.8862, 0.8979]	0.7791 [95% CI: 0.7712, 0.7868]	0.8909 [95% CI: 0.8849, 0.8966]	0.8688 [95% CI: 0.8624, 0.875]
**Kappa**	0.7804	0.7843	0.558	0.7817	0.9003
**Sensitivity**	0.8750	0.9092	0.8172	0.9132	0.8370
**Specificity**	0.9054	0.8749	0.7407	0.8684	0.8477
**Pos Pred Value**	0.9032	0.8799	0.7605	0.8749	0.8477
**Neg Pred Value**	0.8779	0.9053	0.8008	0.9084	0.8928
**Prevalence**	0.5020	0.5020	0.5020	0.5020	0.5020
**Detection Rate**	0.4392	0.4564	0.4102	0.4584	0.4519
**Detection Prevalence**	0.4863	0.5187	0.5394	0.5239	0.5331

**Balanced Accuracy**	0.8902	0.8921	0.7789	0.8908	0.8687
**P-Value [Acc > NIR]**	<2.2e-16	<2.2e-16	<2.2e-16	<2.2e-16	< 2.2e-16
**No Information Rate**	0.502	0.502	0.502	0.502	0.502

Also, ROC for RF was 95.74%, DT was 95.56%, ANN was 85.37%, SGBM was 95.77, and SVM was found to be 92.45%. See below.

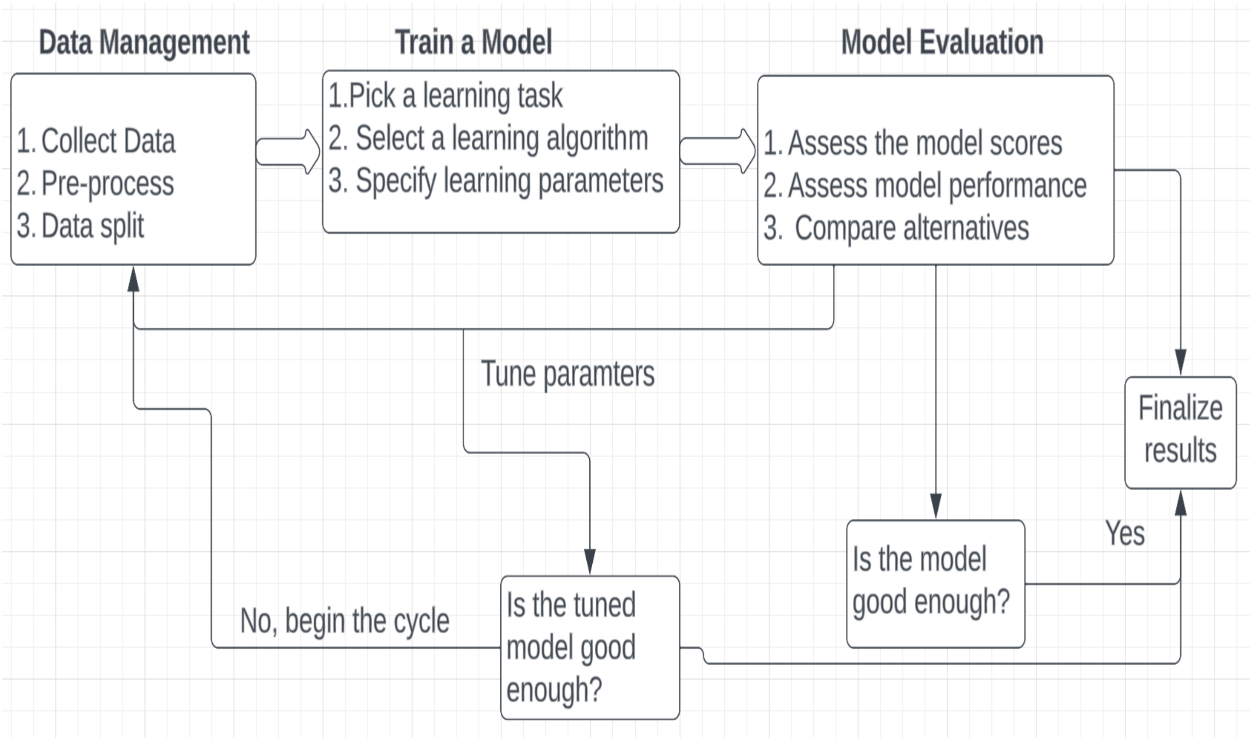

Figure 1: An ML schema

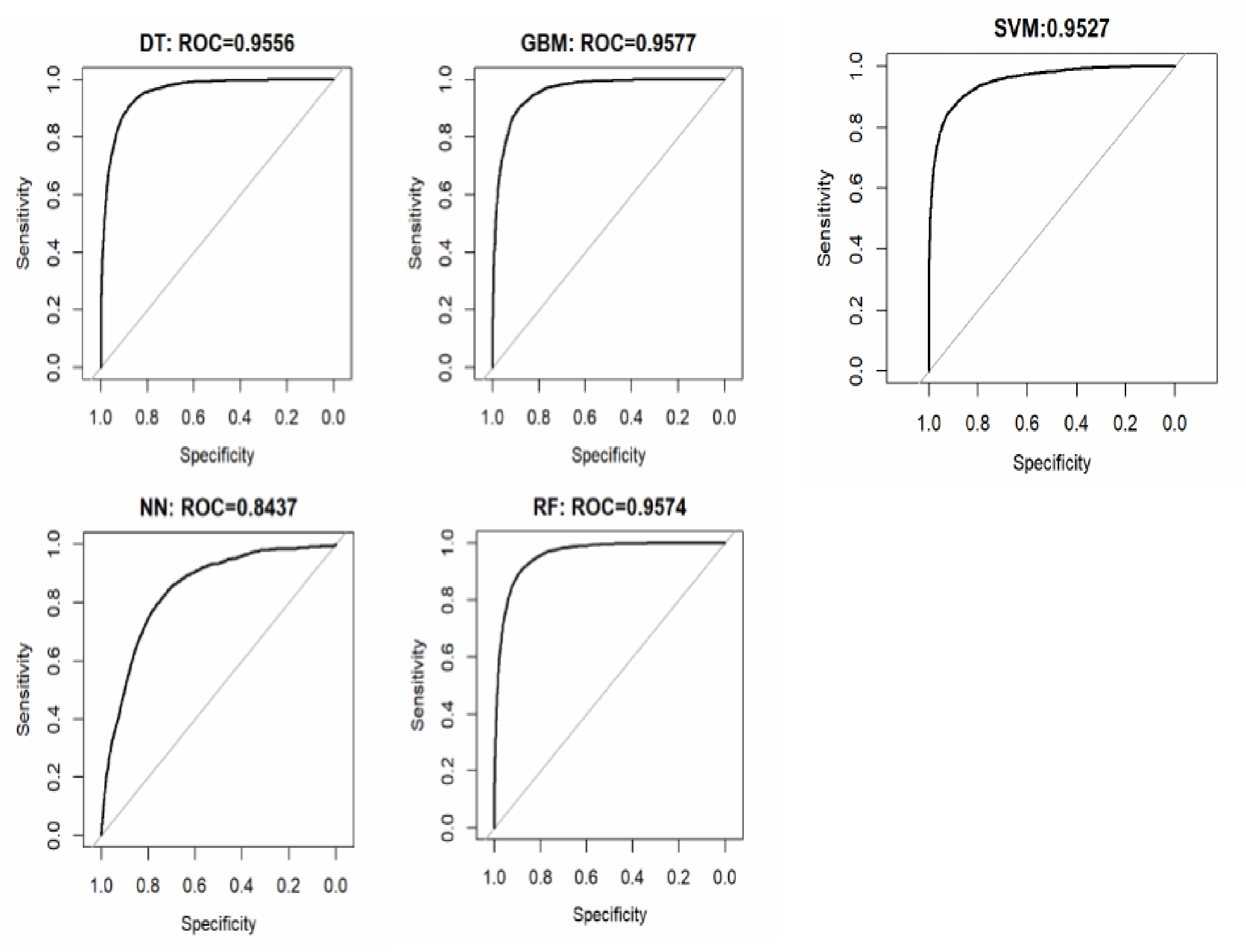

Figure 2: Receiver Operating Characteristics for various ML models

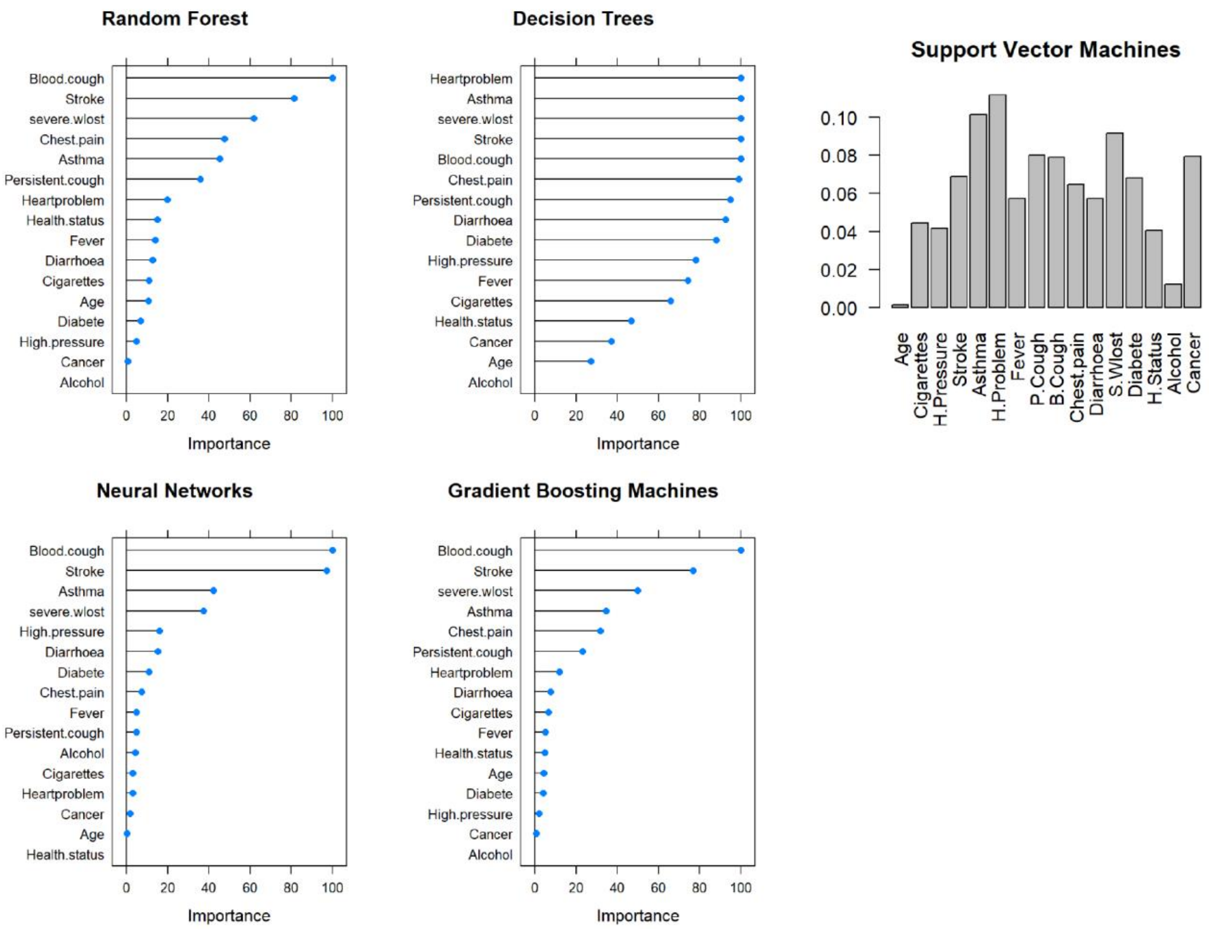

Figure 3: Variable importance plot for the predictors/features
