# Supplementary material for: Predicting and improving diagnosis of tuberculosis outcomes in South Africa using machine learning techniques
Source: PLOS Glob Public Health. 2025 Nov 7;5(11):e0004674. doi: 10.1371/journal.pgph.0004674 (PMC12594327; doi:10.1371/journal.pgph.0004674)
Supplement: Attachment — Submitted filename: Response to Reviewers_TB_13_3_2025 (1).docx [file pgph.0004674.s004.docx]

**INTRO**

A big thank you to the editors for their patience in allowing us to address the various but crucial concerns raised by the reviewers. We appreciate the beneficial comments from the reviewers, which have helped improve the paper.

**GENERAL COMMENTS**

Please use the space provided to explain your answers to the questions above. You may also include additional comments for the author, including concerns about dual publication, research ethics, or publication ethics. (Please upload your review as an attachment if it exceeds 20,000 characters)

Reviewer #1: I have reviewed the original manuscript as submitted and given feedback with minor revision requests in the attached reviewed PDF copy. Overall, I find the study relevant and in compliance with the PLOS Global Public Health Journal publication standard and requirements. The statistical analysis described by the authors was conducted correctly with sound results, discussion, and conclusion sections.

Reviewer #2: The study titled “Predicting Tuberculosis (TB) with Machine Learning Approaches: An Effort to Improve Tuberculosis Diagnosis in South Africa” investigates the feasibility of using feature-based machine learning algorithms for the prediction and diagnosis of TB in South Africa. The authors compare five feature-based machine learning (ML) techniques and assess a variety of performance metrics. They also compute the most important feature predictors that contribute to each of the ML model’s predictions. The authors found that ensemble, tree-based ML methods (RFs, SGBMs) achieved the highest overall predictive performance (in terms of accuracy, sensitivity, specificity, and AUROC), though all five ML methods achieved > 70% accuracy.

Prior literature on this topic does not utilize data from low-resource settings, instead focusing on using advanced medical interventions (e.g. chest X-rays, pharmaceutical solutions) to assess the success of machine learning and deep learning in predicting TB. This work uses only non-invasively collected, self-reported personal information to predict the presence of TB.

This study’s primary contribution is its evaluation of ML for TB prediction in the South African patient population as a low-resource real-world use case. However, its applicability to other countries and regions outside of this context is limited. The high performance of the feature-based ML methods and consistency in top contributing features to prediction across these methods strengthens the study’s conclusion. The authors include a ranking of top contributing features to the prediction, which assists in understanding the model’s predictive behavior and alignment with clinical knowledge. **However, this work’s conclusions is weakened by its lack of sufficient methodological details regarding rigorous evaluation, which should be clarified before considering acceptance.**

Major issues
Comment #1: 1. The authors reference other literature on **using machine learning to predict TB, though prior work focuses on clinical data prevalent in high-resource medical settings (e.g. chest X-rays). The authors should comment on the availability of other feature-based machine learning studies that aim to predict/diagnose TB or, if no such work is currently available, mention this gap.**

**Response #1:**

Thank you very much for your attention and input. We have included this suggestion in the manuscript

**Comment #2**
2. When referring to the hyperparameter grid search procedure (lines 240, 252, 284-285), clearly state which data subset/split was used to compute the accuracy metric used to select the best hyperparameter configuration. Standard practice computes it as an average over 5-fold cross validation folds, but please clarify. Lines 267-270 in the SGBM section defines 5-fold cross-validation. It states that the training data are split into five equal-sized subsets, but there is no mention of how these subsets are used to train/evaluate the subsequent models.

**Response #2**

**Cross-validation procedure**: We split the training data into five equal-sized folds. For every iteration step, we used four subsets to train the model and the remaining for validation. We repeated this five times to ensure that each subset served as the validation set once. We have updated the manuscript accordingly.

**Hyperparameter selection:** The accuracy of the metric used to choose the ideal hyperparameter configuration was calculated as the average of the validation accuracy over the five-cross-validation subsets. This ensured that the selection accounted for variance across different data subsets.

Comment #3
3. ANNs can have any number of hidden layers, not just three (line 245); the authors reference including the both the number of hidden layers and the number of neurons per hidden layer as tunable hyperparameters. How was the hyperparameter search conducted while varying the number of hidden layers and the number of neurons per hidden layer? Was the latter held constant while adjusting the former? Additional details on the specifics should be included.

Response #3

Thank you very much for your attention to these details. To clarify, our hyperparameter tuning was based on two criteria: the number of neurons in the hidden layer and the regularization parameter. We used one hidden layer as a constant.

 **Number of neurons in the hidden layer (size):** We tested four values: (5, 10, 15, and 20).

 **Regularization parameter (decay):** We tested four values: (0.1, 0.01, 0.001, and 0.0001)

The number of hidden layers was fixed at one and did not vary during the tuning, while the rest of the parameters (decay and number of neurons) were allowed to vary using a grid-search approach. The best hyperparameter combination was selected based on **5-fold cross-validation**, using the mean validation accuracy across folds as the selection criterion

**Comment #4**
4. Which layers of the ANN had the sigmoid activation function applied to the outputs?

**Response # 4**

Thank you very much. The model setup for the sigmoid activation function was applied as follows;

1. For the hidden layer, the sigmoid was applied by default by the nnet model to introduce non-linearity.
2. For the output layer; for the binary classification as our case, the sigmoid function was applied to the output layer, to producing the values between 0 and 1 for the predicted probabilities.

**Comment #6**
Accuracy was reported with a 95% confidence interval. How was this interval computed? Were models re-trained on the full 70% training set and evaluated on the 30% held-out test set with bootstrapping? The confusion matrix and other metrics indicate no measure of variance, reported with a single value evaluated on the 30% held-out test set.

**Response #6**

Thank you for your thoughtful comment regarding the computation of the 95% confidence interval for accuracy and the reported performance metrics.

To clarify:

1. The 95% confidence interval for the accuracy was calculated on the 5-fold cross-validation (as detailed in the paper). The accuracy was reported for every fold, and the CI was estimated by bootstrapping these cross-validation accuracies. This made it possible to evaluate the variability of the model's performance across different partitions of the training data.
2. The model was not retrained on the complete 70% training set and then assessed on the 30% held-out test set for the CI. Instead, the performance metrics were derived by cross-validation during the hyperparameter tuning. The test set (30%) was used only for the final model assessment.
3. The confusion matrix and other metrics (e.g., precision, recall, F1 score, etc.) were calculated on the 30% held-out test set. We presented a single evaluation metric, and we acknowledge the lack of variance of these metrics, which is recognized as a limitation of the study.

Comment #7
How were the top/bottom predictors for each of the ML techniques computed?

Response #7

We calculated for variable for each model differently. For tree-based models (e.g., DT, RF, etc.), we used mean decrease in impurities (how the variable contributes to a decrease in split noise during the recursive portioning). For the Neural network, we used a mean reduction in the variable weights according to a specific input to the hidden layer based on the Garson Algorithm. By default, the absolute weights are normalized into 0-100% scale. The SVM was based on recursive feature elimination method whereas the gradient boosting machine was based on a weighted sum of how the variable reduced the log loss and the number of times a variable is used in the tree splitting process.

Minor issues

Comment #8
1. Other more robust methods to assess multicollinearity may be used, like the variance inflation factor (VIF) which calculates how much the variance of a regression coefficient is inflated due to correlations with other features. This can be more robust because it considers more than just pairwise relationships between the features.

Response #8

Yes, we reconducted variance inflation factor. We first fitted a generalized linear model, and then calculated the VIF from that. We did not find any significance inflation by any of the variable we used for the study (<5).

Comment #9
2. Some broad terms require more grounding tomake them relevant for a technical audience (e.g. generalizability line 223, model adequacy line 289, model reliability line 289).

Response #9

Thank you, we have addressed in the manuscript

Comment #10
3. Please clarify lines 251-252: “The desired parameters for our model are for the 10-end superscript to the minus 4-end superscripts.”

Response #10

Thank you for this. We have clarified to make it more readable in the main document

Comment #11
4. NPV excluded from the equation definitions (lines 292-295).

#Response 11

Thank you. We have addressed in the main document

Comment #12
5. TP and FP represent the count of positive cases, not the rate (TPR, FPR).

Response #12

You are right. Thank for this correction. We have corrected it in the manuscript

Comment #13
6. Please clarify that AUC values refer to the area under the ROC in the main text.

Response #13

Thank you for this addition. We have revised in the manuscript

Comment #14
7. Background information on the Kappa metric, detection rate, detection prevalence, balanced accuracy would be helpful to include.

Response #14

You are definitely right. It gives a complete picture of the metrics. But we previously included, not sure if it was an oversight. Thank you.

Comment #15
8. Accuracy > NIR was not reported for all ML techniques (SGBM, SVM excluded) in the main text.

Response #15

Thank you for your concern. Due to the severe class imbalance in the test datasets, relying on the accuracy heavily could result in erroneous conclusion. Therefore, we rather calculated for the f1-score which was previously not done. We used that in combination with the sensitivity and accuracy for the assessment instead of relying on the accuracy metrics.

Comment #16
9. DTs should not be referenced as ensemble models in lines 377-378.

Response #16

Thank you for drawing our attention to this. We have addressed this in manuscript

Comment #17
10. Relative feature importance values for SVMs are not included in the main text. Please include them for completeness.

Response #17

Thank you, we have looked into this

Comment #18
11. Table 2: the prediction and reference labels should be labeled as non-TB and TB instead of 0 and 1.

Response #18

Addressed. Thank you.

Comment #18
12. Table 3: the 95% CI label for DTs is excluded.

Comment #19
13. Table 3: ROC values are listed in the text, but should be included in Table 3 as a separate row.

Response #19

Thank you. We have addressed.

Comment #20
14. Figure 2: axes are labeled as sensitivity and specificity but to maintain consistency with the manuscript, should be TP and FP. Keep in mind that these plots are more commonly presented with true positive rate (TPR) and false positive rate (FPR).

Response #20

Thank you for this. But since that was the algorithm’s default settings, and happen to be more clinically easier, that’s why we did not modify. However, if you feel that’s still necessary thing to do, we are happy to do so.

Comment #21
15. Figure 3: the relative feature importance (x-axis for RF, DT, ANN, and SGBM) ranges from 0-100 whereas it is presented on the y-axis for SVM with values < 1.0. Please make the orientation of the axes, scaling, and abbreviation methods (ANN instead of NN, SGBM instead of GBM) consistent with the manuscript.

Response #21

Thank you. We have revised

**Reviewer #3:**

The study which focuses on using machine learning as part of the South African National Strategic Plan for tackling HIV, STI's and TB with a particular interest in predicting TB cases in the population.

**Comment #1**
- In using SMOTE and ending with No TB=5,585 and TB=5,541 (approximately 1:1) where the original proportions were for No TB=35,847 and TB = 1,381 (approximately 26: 1) you may have introduced introducing biases that may have better been handled with SMOTE proportions potentially of the order of 4:1 or even matching No TB to TB before training (this may also go to the order of 4:1).
- When testing/validation was the testing/validation set separated from the original population before applying SMOTE? To avoid testing SMOTE trained data on SMOTE test data in which case the testing/validation test would not be representative of the truth in the population

- Considering the national strategic plan, in what stage of the process, would this machine learning approach fit in for South Africa? Also what part of the current strategy does this approach outperform? And what are the metrics for comparing machine learning to such a step?

**Response #1**

You are absolutely right. Using a strict 1:1 balance may introduce biases in the estimate. And sure! You made a very crucial observation with respect to “SMOTING” the test set. We rechecked, and that error was indeed made. We have recalibrated the model, and although the accuracy remained relatively high, we had to evaluate for another metric, like the f1 score, which had previously been undone. Also, since there was an imbalance in the sensitivity and specificity trade-off, we used AUC to plot the performance to account for this severe class imbalance in the test datasets. We actually made numerous sensitivity tests ranging from class weighting (on top of the trained data resampling) to use, for example, “balanced random forest” for the model training, to splat scaling, etc., and yet, the class imbalance still played a role in the test set evaluation even after numerous probability threshold adjustment. What has been reported currently appears to be the ideal balance we could achieve. We have added some notes to the limitation of the study section. If you have other suggestions for further improvement, we will be happy to do so!!!!

Although our ML approach has some inherent limitations, it may be helpful for the screening and early case detection stages of the South Africa Strategic Plan for TB. ML can help to emphasize higher-risk individuals proactively rather than through symptoms-based screening. The effectiveness of the ML for such a use case can be evaluated using f1-score, sensitivity, specificity, and AUC-ROC. Hopefully we have addressed your concerns to your expectation.

**Comment #2**
- Line 10-133 . Consider rewording the sentence. Its meaning is unclear. "While several strategic targets by the WHO in 2000 and 2015 have achieved their goal of halving TB (14), in the South, the confluence of TB, HIV/AIDS and increasing cardiometabolic diseases are achieving this target challenging (15)."
**Response #2**

Thank you very much. We have revised

**Comment #3**
- The definition is reversed in 188: "High blood pressure was defined as a systolic pressure of >90mm/hg and a diastolic pressure of 140mm/hg" it should be systolic >= 140mm/Hg and diastolic >= 90mm/Hg.

**Response #3**

Thank you for drawing our attention to this error. We have addressed this.

**Comment #4**
- Line 199 should read "...wrongly spelled values...". Also can you explain what you mean by wrong data structures?

**Response #4**

Thank you for this correction. By wrong data structure, we meant the format. Like a data which is treated as numeric when it should be a factor. We have changed structure to format to clear the confusion.

**Comment #5**
- From line 387 please include the name of the authors and year along with the reference "For example, studies, including the work of (5), showcase the enhanced accuracy of deep learning in classifying X-ray images of potential TB patients, facilitating early detection and subtype differentiation. Furthermore, (6) explored TB drug resistance profiles using machine learning, guiding targeted treatment strategies. Additionally, (7) employed a progressive resizing approach to train models for automatic TB diagnosis, integrating fine-tuned Normalisation-Free Networks (NFNets) and the Score-Cam algorithm to highlight specific regions in chest X-rays for detailed inference."

**Response #5**

Thank you very much for pointing this out. We have addressed this in the manuscript.

**Comment #6**
- Bias: How does the target variable take care of old TB cases, considering the question focuses on the last 30 days?

**Response #6**

This was an error on our part. The full questions were: “We were previously told that you have Tuberculosis / TB; is this correct?” “Have you ever been told by a doctor, nurse, or health care professional that you have Tuberculosis / TB?” “Are you currently taking medication for Tuberculosis / TB?” and “Do you still have Tuberculosis / TB?”.

**Comment #7**
- Is there a reason you prefer in your discussion to focus on accuracy over F1 scores, considering accuracy is generally considered less reliable than F1 scores?

**Response #7**

You are absolutely right about this. It’s because of the error we made previously by using the “SMOTED” test set; we had relatively balanced metrics, so we believed the accuracy would not have been significantly different from the F1 score. However, after we revised and recalibrated the model with the original test set, we now placed more emphasis on the F1 score and the sensitivity due to the severe class imbalance in the test datasets. Again, thank you for these crucial highlights.

6. PLOS authors have the option to publish the peer review history of their article ([what does this mean?](https://track.editorialmanager.com/CL0/https:%2F%2Fjournals.plos.org%2Fglobalpublichealth%2Fs%2Feditorial-and-peer-review-process%23loc-peer-review-history/1/010f019465844301-1f8944b8-64fa-49d2-b9eb-3bec58e296a9-000000/5yXcBsBjjgG-3O7qGyPjvfuDmstq4CscXAtCVEDevgU=193)). If published, this will include your full peer review and any attached files.


**Do you want your identity to be public for this peer review?** If you choose “no”, your identity will remain anonymous but your review may still be made public.


For information about this choice, including consent withdrawal, please see our [Privacy Policy](https://track.editorialmanager.com/CL0/https:%2F%2Fwww.plos.org%2Fprivacy-policy/1/010f019465844301-1f8944b8-64fa-49d2-b9eb-3bec58e296a9-000000/bGlbqzZ9uZ00xzhwqW1fXlctK5daEJveVSXGNeyTCqg=193).

Reviewer #1: **Yes:** Emmanuel Ndubuisi Egbo

Reviewer #2: No

Reviewer #3: No
