# Supplementary material for: Predicting and improving diagnosis of tuberculosis outcomes in South Africa using machine learning techniques
Source: PLOS Glob Public Health. 2025 Nov 7;5(11):e0004674. doi: 10.1371/journal.pgph.0004674 (PMC12594327; doi:10.1371/journal.pgph.0004674)
Supplement: Attachment — Submitted filename: Response to reviwers_9_4_2025.docx [file pgph.0004674.s005.docx]

**RESPONSES TO REVIEWER COMMENTS**

**General Comment:** Reviewer #4: The paper explores an important topic in the field of public health and shows that machine learning techniques can be useful in diagnosing tuberculosis. However, to increase the validity of the results, it is necessary to provide more details about the methods and statistical analyses.

**General Response:** Thank you very much for all your effort in making these important suggestions. To the best of our ability, we have addressed these comments satisfactorily.

**Comment #1:** 1-It would be better to discuss the research background related to the use of machine learning in tuberculosis diagnosis further.

Response #1: We have updated the manuscript, adding further studies on TB and ML. Kindly see page 3 line 71 to page 4 line 81. Thank you very much.

**Comment #2:** 2-Details on data preprocessing, including missing data handling and normalization, are not provided.

**Response #2:** Thank you for your time, and willingness to improve our paper. However, we have a section that handles the concerns you have already raised. Kindly see page 9 line 207 to page 10 line 223. Again, thank you for your effort.

**Comment #3**- The exact hyper-parameter settings for each model are not known.

**Response #3:** Thank you, but we think there was an oversight. We have been specific about the exact hyperparameters that were finally used for training the model. For Random Forests (RFs) and Decision Trees (DTs), kindly see pages 10, line 236, to 11, line 265.

**Comment #4**-providing confidence interval values for accuracy, sensitivity, and specificity can help to better evaluate the results.

**Response #4:** Thank you very much for your attention to our paper. We computed a 95% confidence interval for accuracy. Kindly see Table 3. However, we could not compute for the sensitivity and specificity as we were limited in our ability to do so due to the algorithmic setup. Since caret packages typically use accuracy and kappa for parameter decisioning, the 95% CI is only computed for accuracy. I believe this constraint explains why all these studies (1–4) did not include 95% Cis for the metrics. We acknowledge the lack of variance in these metrics, which is recognized as a limitation of the study.

**Comment #5-**Statistical comparisons between model performance have not been performed; using statistical tests could reveal significant differences.

**Response #5:** Thank you very much for drawing our attention to this. We compared the accuracies and kappa for the models using the Friedman rank sum test. We used the non-parametric counterpart to avoid making assumptions of the distribution, which is more ideal. Again, we could test this on the accuracies and kappa as the caret package is customarily set for these parameters. Still, we believe the inclusion of the kappa helps account for the class imbalance inherent in the data. We have added this to the paper. See page 11, lines 243-244 & page 17, lines 395-396.

**Comment #6**-Further explanation is needed about the reasons why the RF and DT models perform better than other models.

**Response #6:** Please see page 21, lines 480 to 489. We have discussed why tree-based models performed better, stressing SGBMs and RF, and the implications thereof. Kindly let us know if there is further information you feel is worthwhile. We will be happy to provide it.

**Comment #7**- It would be helpful to discuss the limitations of the study, such as the generalization of the results to other populations.

**Response #7**: Please note that we have included the study's limitations from page 21, line 491, to page 22, line 514. We discussed generalizability-related demographic biases, gender representativeness, extreme class imbalance, trade-offs made, and their implications. We appreciate your effort in making suggestions in these critical components.

**References**

1.	Tang N, Yuan M, Chen Z, Ma J, Sun R, Yang Y, et al. Machine Learning Prediction Model of Tuberculosis Incidence Based on Meteorological Factors and Air Pollutants. Int J Environ Res Public Health. 2023 Jan;20(5):3910.

2.	Fayaz SA, Babu L, Paridayal L, Vasantha M, Paramasivam P, Sundarakumar K, et al. Machine learning algorithms to predict treatment success for patients with pulmonary tuberculosis. PLOS ONE. 2024 Oct 16;19(10):e0309151.

3.	Kalhori SRN, Zeng XJ. Evaluation and Comparison of Different Machine Learning Methods to Predict Outcome of Tuberculosis Treatment Course. 2013 Jul 31 [cited 2025 Apr 9];2013. Available from: http://www.scirp.org/journal/PaperInformation.aspx?PaperID=35397

4.	Jamal S, Khubaib M, Gangwar R, Grover S, Grover A, Hasnain SE. Artificial Intelligence and Machine learning based prediction of resistant and susceptible mutations in Mycobacterium tuberculosis. Sci Rep. 2020 Mar 26;10(1):5487.
