# Supplementary material for: Predicting and improving diagnosis of tuberculosis outcomes in South Africa using machine learning techniques
Source: PLOS Glob Public Health. 2025 Nov 7;5(11):e0004674. doi: 10.1371/journal.pgph.0004674 (PMC12594327; doi:10.1371/journal.pgph.0004674)
Supplement: Attachment — Submitted filename: Response to Reviewers_21_6_2025.docx [file pgph.0004674.s006.docx]

**Reviewer #4:**

This manuscript presents a timely and relevant study applying multiple machine learning (ML) algorithms to improve tuberculosis (TB) diagnosis in South Africa. The study is methodologically sound in many aspects and contributes meaningfully to public health research in low-resource settings. The following are suggested for improvement:

**Comment #1**

Although the authors mention dataset limitations, the lack of subgroup analysis by gender or race—two key epidemiological factors in TB—is a limitation that must be better discussed.

**Response #1**

We fully concur that race and gender constitute critical epidemiological variables in tuberculosis research. Regrettably, our dataset lacked sufficiently granular or comprehensive race-based information necessary to facilitate meaningful subgroup analysis. Concerning gender, although it was recorded, the distribution within our overall datasets exhibited a significant imbalance. Undertaking further stratified analysis under such circumstances would compromise the statistical reliability of the results, particularly in terms of accuracy, sensitivity, and generalizability. Given analogous conditions, partitioning based on gender proved suboptimal. Nonetheless, considering that the male-to-female ratio in South Africa is nearly equal (approximately 0.954), we assert that the results are highly representative of these groups.

We have explicitly acknowledged these limitations in the revised Discussion section, underscoring their implications for fairness and external validity. Furthermore, we emphasize the importance of future studies in collecting and reporting more demographically balanced data to facilitate robust subgroup analyses, which are essential for equitable algorithm development and deployment in the field of public health. All revisions are marked in green in the revised paper. Thank you.

**Comment #2**

The paper would benefit from further reflection on how these models could be integrated into actual clinical or public health workflows. What are the next steps for operationalization?

**Response #2**

We sincerely appreciate your bringing this matter to our attention. We have integrated it into the revised manuscript, and all modifications are indicated in green.

**Comment #3**

Lack of baseline comparison with logistic regression:

A conventional baseline model (e.g., logistic regression) should be included for comparison to assess whether ML models truly provide superior performance. All revisions are highlighted in green.

**Response #3**

We have integrated this into our manuscript. Thank you.
