# Supplementary material for: Predicting and improving diagnosis of tuberculosis outcomes in South Africa using machine learning techniques
Source: PLOS Glob Public Health. 2025 Nov 7;5(11):e0004674. doi: 10.1371/journal.pgph.0004674 (PMC12594327; doi:10.1371/journal.pgph.0004674)
Supplement: Attachment — Submitted filename: Response to the reviewer_v4.docx [file pgph.0004674.s007.docx]

**GENERAL COMMENT**

Reviewer #4: The manuscript is scientifically sound and fits well with the scope of the journal. It would be better to pay attention to the following:

**GENERAL RESPONSE:** Thank you very much for your time and effort. We have addressed all comments to the best of our ability. All changes are highlighted in blue in the main manuscript.

**SPECIFIC COMMENTS**

**Comment #1**

1-Extensive technical details are not necessary. Consider summarizing the model architecture and parameters in the supplementary material and focusing more on interpretability in the main text. It would be useful to discuss how these models are understood or used by healthcare workers in practice

**Response #1:** Thank you very much for your dedicated time. We have removed all details to the supplementary file now.

**Comment #2**

2-The ethical implications of deploying ML models in healthcare, particularly with low specificity, should be further elaborated. Misclassification in TB screening may lead to over-treatment or missed diagnoses.

**Response #2:** We have expanded this concern in the limitation section of the manuscript. Thank you.

**Comment #3**

3-Given the role of HIV in TB comorbidity in South Africa, the absence of this variable in the dataset is a major limitation. This should be clearly emphasized and considered in the discussion

**Response #3:** We have included this as part of the limitations of the study. Thank you.

**Comment #4**

4-Interpret the results with foresight and generalizability

**Response #4:** We have included this important suggestion in the main manuscript. Thank you.

**Comment #5:** 5-Include a figure summarizing model performance metrics side by side

**Response #5:** We have created a plot for all the relevant performance metrics against the six models. Thank you.
