# Supplementary material for: Predicting and improving diagnosis of tuberculosis outcomes in South Africa using machine learning techniques
Source: PLOS Glob Public Health. 2025 Nov 7;5(11):e0004674. doi: 10.1371/journal.pgph.0004674 (PMC12594327; doi:10.1371/journal.pgph.0004674)
Supplement: Attachment — Submitted filename: Response to the reviewer_v5.docx [file pgph.0004674.s008.docx]

**General Comments**

Reviewer #4: The study is methodologically sound in many aspects and contributes meaningfully to public health research in low-resource settings, Fortunately, the requested improvements have been largely implemented But the following still need to be addressed:

**General Response**

Thank you for your feedback. All changes in the manuscript are highlighted in green color.

**Comment #1**
1- Although the authors mention dataset limitations, the lack of subgroup analysis by gender or race—two key epidemiological factors in TB—is a limitation that must be better discussed.

**Response #1**

Thank you for this. However, we acknowledged this limitation based on your previous comments. We addressed that as part of the “Limitations of the study section”. Please refer to page 20, lines 487 to 503. While we did not have a race variable in our data (which we highlighted in the paper), stratifying the analysis according to gender could weaken the model’s predictive power due to high data imbalance. We stated this in our earlier revisions. Kindly refer to the page and lines indicated.

**Comment #2**
2-How were the top/bottom predictors for each of the ML techniques computed?

**Response #2**

We have included all these in the methodology already. You asked us to move the details to the supplementary table. Kindly see the supplementary material two for these details. Thank you.

**Comment #3**
3-Considering the national strategic plan, in what stage of the process would this machine learning approach fit in for South Africa? Also, what part of the current strategy does this approach outperform? And what are the metrics for comparing machine learning to such a step?

**Response #3**

Thank you. We have included this additional information in the manuscript. See page 20 line 474 and page 21 page 482.
